# *In Vitro* Determination of Respimat^®^ Dose Delivery in Children: An Evaluation Based on Inhalation Flow Profiles and Mouth–Throat Models

**DOI:** 10.1089/jamp.2014.1166

**Published:** 2016-02-01

**Authors:** Deborah Bickmann, Wolfgang Kamin, Ashish Sharma, Herbert Wachtel, Petra Moroni-Zentgraf, Stefan Zielen

**Affiliations:** ^1^ Pharmaceutical Physics Laboratory, Boehringer Ingelheim Pharma GmbH and Co. KG, Ingelheim, Germany.; ^2^ Children's Hospital, Evangelisches Krankenhaus Hamm, Germany.; ^3^ Clinical Pharmacokinetics, Translational Medicine and Clinical Pharmacology, Boehringer Ingelheim Pharma GmbH and Co. KG, Ingelheim, Germany.; ^4^ Children's Hospital, Allergology, Pneumology and Cystic Fibrosis, Goethe-University, Frankfurt am Main, Germany.

**Keywords:** children, fine particle-fraction, inhalation breathing pattern, inspiratory flow, lung deposition, Respimat^®^ Soft Mist™ Inhaler

## Abstract

***Background:*** Aerosol therapy in young children can be difficult. A realistic model based on handling studies and *in vitro* investigations can complement clinical deposition studies and be used to enable dose-to-the-lung (DTL) predictions.

***Methods:*** Predictions on dose delivery to the lung were based on (1) representative inhalation flow profiles from children enrolled in a Respimat^®^ handling study, (2) *in vitro* measurement of the fine-particle DTL using mouth–throat models derived from nuclear magnetic resonance/computed tomography (NMR/CT) scans of children, and (3) a mathematical model to predict the tiotropium DTL. Accuracy of the prediction was confirmed using pharmacokinetic (PK) data from children with cystic fibrosis enrolled in a phase 3 clinical trial of tiotropium Respimat with valved holding chamber (VHC).

***Results:*** Representative inhalation flow profiles for each age group were obtained from 56 children who successfully inhaled a volume >0.15 L from the Respimat with VHC. Average dimensions of the mouth–throat region for 38 children aged 1–<2 years, 2–<3 years, 3–<4 years, and 4–<5 years were determined from NMR/CT scans. The DTL from the Respimat plus VHC were determined by *in vitro* measurement and were 5.1±1.1%, 15.6%±1.4%, 17.9%±1.5%, and 37.1%±1.8% of the delivered dose for child models 0–<2 years, 2–<3 years, 3–<4 years, and 4–<5 years, respectively. This provides a possible explanation for the age dependence of clinical PK data obtained from the phase 3 tiotropium trial. Calculated *in vitro* DTL per body mass (μg/kg [±SD]) were 0.031±0.014, 0.066±0.031, 0.058±0.024, and 0.059±0.029, respectively, compared to 0.046 in adults. Therefore, efficacy of the treatment was not negatively impacted in spite of the seemingly low percentages of the DTL.

***Conclusions:*** We conclude that the combination of real-life inhalation profiles with respective mouth–throat models and *in vitro* determination of delivered DTL is a good predictor of the drug delivery to children via the Respimat with VHC. The data provided can be used to support data from appropriate clinical trials.

## Introduction

Many respiratory diseases in adults originate in childhood, with as much as 60% of asthma in adults being traced to the childhood years.^(1)^ Furthermore, in the United Kingdom, 25% of all pediatric outpatient attendances, and 35% of pediatric primary care consultations, are due to significant respiratory morbidity.^(1)^ These numbers reflect the importance of treating respiratory disease in early childhood. In adults with respiratory diseases, inhalation therapy is most commonly used for delivering treatment with bronchodilators and corticosteroids to relieve symptoms and prevent exacerbations.^(2)^ A variety of inhaler devices are currently available for patients with respiratory diseases. The choice of device is an important consideration because it can influence patients' adherence to treatment and, thus, potentially affect the long-term outcome.^(3–6)^

Pressurized metered-dose inhalers, the most frequently prescribed devices worldwide, allow direct delivery of a drug to the lung;^(7)^ however, because many patients fail to use pressurized metered-dose inhalers correctly, these devices are limited by the necessity of a good coordination of patient inspiration and inhaler activation. However, in a pediatric population, the use of a valved holding chamber (VHC) is recommended in the joint European Respiratory Society and International Society for Aerosols in Medicine task force report 2011.^(8)^ Dry powder inhalers, which do not require active coordination, are likewise limited by a sufficient inspiratory flow, which is difficult to achieve by children.^(7)^

A recently introduced inhaler, Respimat^®^, the first “Soft Mist™ Inhaler” (SMI) (Boehringer Ingelheim, Ingelheim, Germany), releases the drug solution as a slow-moving aerosol, so that lung deposition is both improved and reproducible.^(2)^ Its aerosolized spray disperses slowly (0.8 m/s at 10 cm distance), is long lasting (1.5 s), and has a high fine-particle fraction (66% of particles<5.8 μm).^(9)^ Active inhalers can be used by children, and pressurized metered-dose inhalers can be equipped with a VHC in order to facilitate the coordination between the release of the aerosol and the inhalation maneuver. Recent handling studies have shown that the Respimat SMI is suitable for use in children.^(10)^ Below the age of 5 years the use of a VHC with face mask facilitates dose administration with tidal breathing and reduces the need for coordination between dose release and inhalation.

There is a need for safety and optimal clinical control of aerosol delivery in children. The inhaler used in combination with the VHC device and inhalation proficiency determines the dose to the lung. However, *in vivo* quantitative determination of the dose delivered to the lung in children is challenging because there are limited possibilities to use pharmacokinetic (PK) methods in young children, as well as ethical restrictions on administering radiolabeled drugs to children for obtaining scintigraphic pictures on lung deposition. Blood sampling in children is also ethically and practically difficult, and urine collection is challenging, especially in young children.^(11)^

Additionally, studies involving the use of inhaled medication in children can be subject to high levels of variability. For inhaled medication, although PK measurements can be used to determine the dose to the lung, they are not considered predictive of efficacy. For these reasons, *in vitro* measurements may be used to obtain experimental data on the dose delivered to the lung in order to support dose selection for clinical trials and to better interpret PK profiles.

The method to determine the dose to the lung using throat models has recently been optimized for adult mouth–throat models,^(12)^ and the current study reports on the prediction of the dose to the lung in children aged under 5 years of age using Respimat with a VHC with face mask.

## Materials and Methods

### Study design

The study was designed to mimic *in vivo* dosing to the lung from an inhaler with VHC in children. The standard United States Pharmacopeia (USP) method^(13)^ was adapted to reflect this. Mouth–throat geometries derived from nuclear magnetic resonance/computed tomography (NMR/CT) scans of children at the Johannes Gutenberg University (Mainz, Germany) were used to create mouth–throat models that could be linked to the Respimat^®^ SMI with VHC and face mask. The Respimat SMI with VHC was connected to an impactor for measuring the fine particle dose to the lung (FPDTL), defined as the mass of particles with diameters<5 μm.

Age group-representative inhalation flow profile patterns from children with a history of cough or wheezing (acquired from the open, observational, two-center handling study of the Respimat SMI with VHC conducted at the Center of Paediatric and Adolescent Medicine at Mainz University Hospital, Germany, and Center of Paediatric and Adolescent Medicine at Frankfurt University Hospital) were used as input for the model.

The model derived was used to predict the DTL for individual inhalation flow profiles and mouth–throat geometries. PK exposure data for Respimat were obtained from a phase 3, 12-week, multinational, multicenter, randomized, placebo-controlled, double-blind, parallel-group study (ClinicalTrials.gov identifier: NCT00737100 and NCT01179347) comparing the efficacy and safety of tiotropium Respimat (5 μg administered as two puffs of 2.5 μg) with placebo once daily on top of usual CF maintenance therapy. The PK data obtained were compared with the predicted values from the model.

### Study population

The handling study (Kamin W et al. JAMPDD, accepted for publication) enrolled 103 children, stratified into four age groups (0–<2 years, 2–<3 years, 3–<4 years, and 4–<5 years), with any respiratory disease, who showed a history of coughing and/or wheezing. For the purpose of the present study, only the children using Respimat SMI with VHC (66 of 103 children) were considered. Of these, 56 children who successfully inhaled a volume >0.15 L were analyzed for the present study.

The study was conducted in accordance with Good Clinical Practice, applicable regulatory requirements, and the principles in the Declaration of Helsinki (1996), and a parent or caregiver for each child provided prior written informed consent. PK measurements (urinary excretion) were obtained for children aged <5 years from a phase 3 trial (ClinicalTrials.gov identifier: NCT00737100 and NCT01179347) that randomized 23 individuals aged <5 years, of whom 15 were randomized to tiotropium 5 μg.

### Materials

The delivery of tiotropium was achieved using the Respimat SMI coupled with VHC (the AeroChamber Plus valved holding chamber Flow-Vu with face mask [Trudell Medical International, London, Ontario, Canada]). Tight connection between the face mask and the model was ensured with a clamp.

### Determination of typical inhalation flow profiles for children

Inhalation flow rates generated by children as a function of time were measured in the handling study by connecting the Respimat and VHC to a pneumotachograph (Masterscope, CareFusion, San Diego, CA) recording the inhalation profiles. Five inhalation cycles with tidal breathing were taken in each age group configuration according to the instruction for the VHC, and a profile that was typical and realistic for each age group was selected.

### Determination of mouth–throat geometries and realistic models in children

**FIG. 1.**
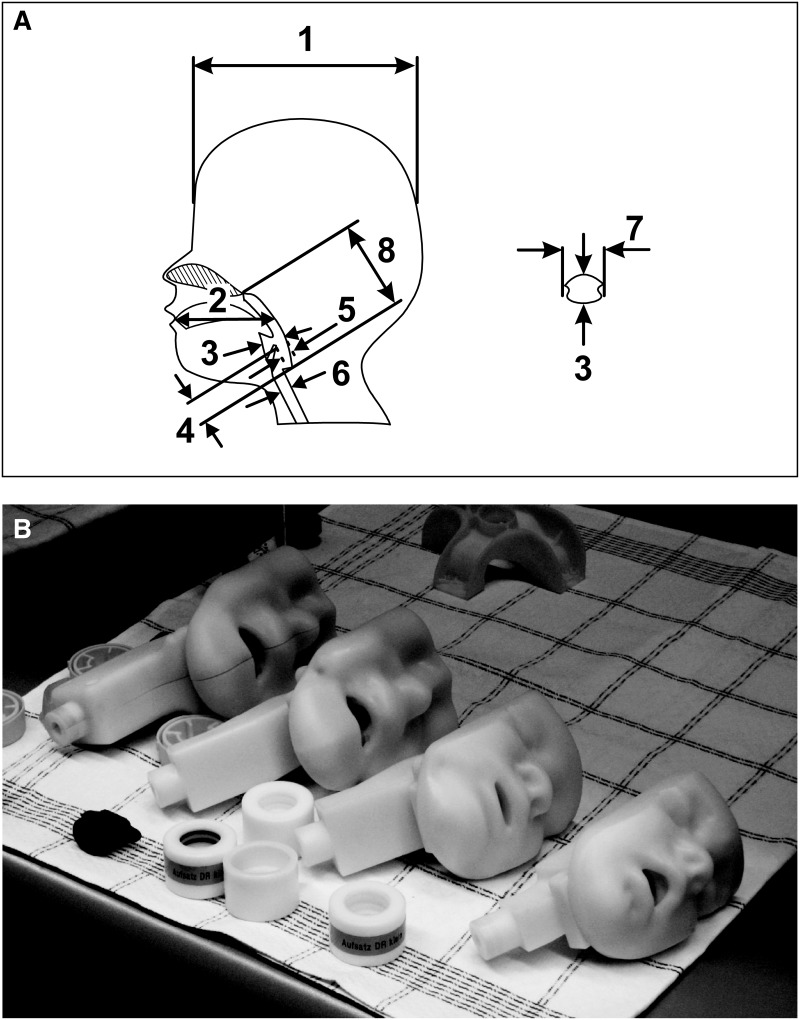
Definition of the characteristic dimensions used for selecting the representative mouth-throat geometries of children **(A)** and the realistic prototype models used **(B)**. From left to right, 4–<5 years, 3–<4 years, 2–<3 years, 1–<2 years age groups.

Mouth–throat geometries were derived from 38 anonymized NMR/CT scans of children with ages equally spaced between 1 and 5 years, performed at the Johannes Gutenberg University, Mainz, Germany. The NMR/CT scans were conducted because of clinical indications and recycled after pseudonymization and institutional review. The NMR/CT data were visualized using Slicer V3.4 (www.slicer.org).^(14)^ The scans were analyzed using the eight defined dimensions depicted in Figure 1A, and this data set was used for selecting typical mouth–throat scans relating to specific age groups (4–<5 years, 3–<4 years, 2–<3 years, 1–<2 years). The selected typical scans were segmented by ITK-SNAP V.1.9.11 (www.itk-snap.org).^(15)^ The resulting Stl files were edited and optimized with ICEM (Ansys, Darmstadt, Germany). Computer-aided design work was performed with Solid Works (Dassault Systems, Solid Works Corporation, Concord, MA). The final realistic mouth–throat models were generated by rapid prototyping using an ABS-type polymer (High Q Prototyping GmbH, Bad Kreuznach, Germany) as depicted in Figure 1B.

### *In vitro* measurement of the drug delivered using predetermined inhalation flow profiles

**FIG. 2.**
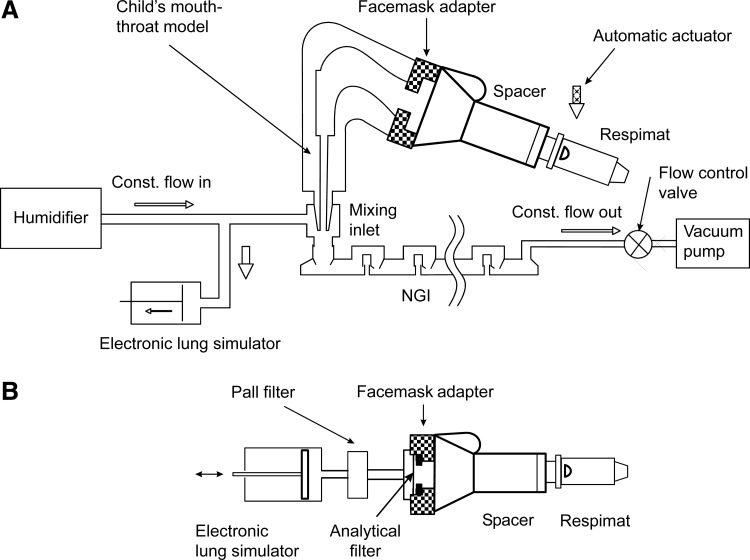
Schematic drawings of the experimental setup using flow profiles for *in vitro* determination of the FPD delivered to the lung setup **(A)** and delivered dose (filter measurement) capturing the total output **(B)**. FPD, fine-particle dose of aerodynamic diameter <5 μm; NGI, Next Generation Impactor, operated at RH>90% from humidifier.

The tiotropium FPDTL was defined as the mass of particles with an aerodynamic particle diameter ≤5 μm and it was determined with a Next Generation Impactor (NGI, MSP Corporation, Shore View, MN). Aerosol delivery from the Respimat and VHC was determined with a setup based on the sampling apparatus for pressurized metered dose inhalers.^(13)^ The rectangular USP inlet was replaced by the mouth–throat model and the face mask of the VHC was fitted to the mouth of the model using a tight face mask adapter (Fig. 2A). Because the breathing pattern through a VHC differs from that through an inhaler, an electronic lung simulator (ASL 5000 [IngMar Medical, Ltd., Pittsburgh, PA]) was included in the experimental setup. The inhalation flow profiles corresponding to the mouth–throat models of the specific age groups were then used to activate the setup through the electronic lung simulator (see Fig. 2 A).

A constant airflow of 60 L/min provided by the humidifier passes through the mixing inlet and is finally sucked through the NGI, where a constant outflow is maintained by a control valve and a vacuum pump. The electronic lung “inhales” air and reduces the inflow to the mixing inlet, while the outflow remains unaffected because of the constant air flow generated by the vacuum pump. In order to balance the inflow and outflow at the mixing inlet, the missing “inhaled” air is drawn into the air vents of the inhaler and passes through the VHC and the throat model. For calibration purposes, a measurement at constant air flow with a rectangular USP inlet was made, with the vacuum pump providing a constant air flow of 60 L/min. The delivered dose was collected with an alternative setup. The dead volume between the filter and the face mask was minimized.

To accommodate the inclusion of the electronic lung, the filter (Whatman^®^ GmbH, Dassel, Germany) was placed directly behind the adapter for the face mask and in front of the tube leading to the electronic lung simulator. This arrangement without a sample collection tube prevents the aerosol from being pumped back into the dead volume without being absorbed into the filter (Fig. 2B).

In both arrangements, the Respimat was equipped with an automatic activator to adjust the exact time intervals between start of the air flow and actuating the inhaler.

In every experimental run, the Respimat was actuated 10 times (by pressing the dose-release button). To provide data on the robustness of the system, Respimat with VHC in respect of differences in flow rate, inhaled volume, and time delay between dose release and inhalation, these parameters were varied. For the experimental investigation, two different constant air flow rates of 5 L/min and 10 L/min were applied. The total volume V being sucked through the inhaler/spacer was investigated for the values of V=1 L and V=0.25 L. Finally data were generated to obtain information about the impact of the delay in seconds (time between the release of the inhaler and the start of inhalation) on the delivered dose.

The amount of drug deposited was quantified by high-performance liquid chromatography (HPLC). All measurements were performed at 23°–25°C and a relative humidity of 50±5%.

### Determination of tiotropium PK from phase 3 trials

The PK of tiotropium was evaluated from urine samples from a subset of patients aged <5 years. Urine sampling at study day 1 and week 12 allowed determination of the amount of tiotropium that was eliminated unchanged in urine at 0–2 hours or 0–4 hours (Ae_0–2_ and Ae_0–4_, respectively) postdose, as well as the fraction of tiotropium dose excreted in urine over 2 or 4 hours). Urine concentrations of tiotropium were determined by a validated HPLC tandem mass spectrometry assay using (D3) tiotropium as the internal standard.^(16)^ The analysis was performed by Nuvisan GmbH (Neu-Ulm, Germany).

### Statistical analyses

Descriptive statistics, including N, arithmetic mean, standard deviation, minimum, median, maximum, arithmetic coefficient of variation (CV), geometric mean, and the geometric CV, were calculated for all fractions of analyte excreted in urine as well as for the PK parameters described above. Statistical significance was assessed by the *t*-test, where appropriate.

### Description of the mathematical model and calculation of the dose to the lung

The model investigates the influence of the inhalation flow profiles of five inhalation cycles on the FPD delivered to the lung. The influence of the additional volume of the VHC and other air ducts on the dose to the lung was also considered. Age dependency of the volume of the upper respiratory tract, as well as the inhaled volume of air was considered, on top of the individual variability documented by the mouth-throat models.

The model takes into account the previous investigation on aerosol delivery from VHCs by Zak et al.^(17)^ Tidal breathing was used for the calculation. The dead space volume (VD),^(17)^ the volume of the VHC (Vch), and the tidal volume (VT) were considered. VT was calculated from the inhalation flow profiles. The delay between release and start of inhalation (Delta2 – s), the average length of the inhalation breaths (Tpulse – s), the breathing frequency (freq – 1/s), the volume inhaled after release of the Respimat within 10 s (V_in_ – L), and the number of breaths recorded within the volume V_in_ (Count – target=5) were derived from the inhalation flow profiles and used to calculate the dose delivered to the lung.

The calculation itself was performed in Visual Basic, as a function macro in Excel (Microsoft, Redmond, WA). The following two steps were considered: (1) the FPD expelled from the VHC was calculated and fitted into the calibration experiments performed with constant flow rate, and (2) the losses in the upper respiratory tract were considered in the way Zak et al.^(17)^ treated “dead space” in their calculation.

In the first step, the following two limiting situations were distinguished: (1) the single breath is considerably smaller than the Vch, and (2) the first breath empties most of the VHC and the remaining four breathing cycles are less important. The decision to perform either one of the two possible different calculations depends on the quotient of the volume per breath (V_in_/count) divided by Vch. If the quotient is smaller than the value b=0.33, then the following calculation is performed:

\begin{align*}FPD_a : &= par_0 \cdot \left[ \mathop \sum
\limits_{n = 0}^4 \left[ {1 \over Vch} \cdot \left( {V_{in} \over
{count}} \right) ^2 \cdot \left( 1 - {V_{in} \over {Vch \cdot
count}} \right)^n \right.\right.\\ & \quad\left. \left.\cdot \exp
\left[ par_1 \cdot  \left( Delta2 + {{Tpluse} \over 2} + {n \over
freq} \right) \right] \right] \right]  + par_2 \tag { \rm Eq. \
A}\end{align*}

if V_in_ is large compared with Vch, then the following equation is used:

\begin{align*}FPD_b : & = par_0 \cdot \left[ \mathop \sum
\limits_{n = 0}^4 \left[ {V_{in} \over {count}} \cdot b \cdot^{
\left[ ( 1 - b ) ^{n \cdot {V_{in} \over {Vch \cdot count}}}
\right] } \right.\right.\\ & \quad \left.\left.\cdot \exp \left[
par_1 \cdot  \left( Delta2 + {Tpulse \over 2} + {n \over freq}
\right) \right] \right] \right]  +  \ par_2 \tag { \rm Eq. \
B}\end{align*}

The parameters that are used in the above formulae have been determined by a least squares fitting procedure within MathCad (PTC, Needham, MA): Vch=0.15 L, par_0_=17.846 μg/L (parameters for PSD estimation with VHC), par_1_=−0.299 1/s (range tested 1.1 to 3.3 μg tiotropium of 5 μg label claim), par_2_=0.991 μg (standard deviation from experiment 0.2 μg), b=0.33 (–).

The constants were determined assuming SI units for the input data, which are given in parentheses following the respective variables: V_in_ (L), Vch (L), count (–), Delta2 (s), Tpulse (s), and freq (1/s).

With regard to formula A applied to low volume breaths: a sum of five breaths was added up, starting with *n*=0. The term V_in_/count is the volume of one single breath. The quotient V_in_/count divided by Vch is the fraction of aerosol volume removed by the first inhalation (*n*=0). The term in parentheses to the power of *n* describes the successive emptying of the VHC volume by following breaths. The exponential decrease was taken into account by multiplying by the exponential term “*par_1_*,” which describes the rate of the decrease, and the time that applies to the respective breath is calculated as a sum of Delta2, Tpulse/2, and n/freq.

The volume of a single breath must be smaller than Vch in order to avoid a negative term in parentheses to the power of n. Therefore, for breathing volumes exceeding at least a third of the Vch, formula B was used. The parameter b represents the fraction of aerosol that is removed from the VHC when a breath is applied. Multiple breaths result in decreasing amounts of aerosol extracted from the VHC. The exponential time dependence is the same as discussed in formula A. It is important to realize that the parameter b is defined between 0 and 1. “b” should not be too large; a value below 0.5 ensures a smooth transition between the two formulae.

The second step of the model calculation is performed in order to account for the dead volume VD of the mouth–throat region: a volume fraction (V_in_ – count*VD) divided by the volume V_in_ is multiplied by the scale factor of 72.8 % and referred to one breath (division by 5, as V_in_ refers to the target of 5 breaths by definition). The difference (V_in_ – count*VD) can approach zero if the VD is large. Negative values may be obtained that are not numerically relevant and they were set to zero.

## Results

### Mouth–throat geometries and models

In order to create realistic mouth–throat models of children according to their specific age groups (4–<5 years, 3–<4 years, 2–<3 years, and 1–<2 years), the mouth and throat of 38 children equally spaced between the ages of 1 and 5 years were scanned by NMR/CT scans. Eight defined dimensions, depicted in Figure 1A, were used to analyze the scans, and the average dimensions for each age group (Table 1) were used to select the most representative realistic mouth-throat models for each specific age group (Fig. 1B). Further details are given in the Supplementary Material (available online at www.liebertpub.com/jamp).

**Table 1.** Average Dimensions Used for Construction of Mouth–Throat Models in Children as a Function of Age Group

*Age group (years)*	n	*Dim 1 (mm)*	*Dim 2 (mm)*	*Dim 3 (mm)*	*Dim 4 (mm)*	*Dim 5 (mm)*	*Dim 6 (mm)*	*Dim 7 (mm)*	*Dim 8 (mm)*	*Total V (L)*
0–<2	10	156.6	53.5	11.6	14.3	6.2	5.8	14.9	47	0.0112
2–<3	8	154.6	58.1	14.5	15.2	5.7	7.2	18.8	48	0.0148
3–<4	9	166.5	64.8	14.6	15.4	9.6	6.2	23.6	45.3	0.0245
4–<5	11	166.6	68.5	15.8	17.1	9.3	6.2	17.4	53.2	0.0398

Dim, dimension; V, volume.

### *In vitro* measurement of the delivered dose using predetermined inhalation flow profiles

A representative inhalation flow profile for each age group was obtained from the handling study. By combining the age-specific realistic throat models with their corresponding inhalation flow profiles, the FPDTL (dose <5.0 μm) from the VHC could be determined. Figure 2 shows the experimental setup used for the *in vitro* determination.

By varying the flow rate, inhaled volume, and time delay between dose release and inhalation parameters, the robustness of the Respimat with VHC system was tested. The three parameters were found to impact on the delivered dose, and consequently FPDTL, with increased delay or reduced volume resulting in a lower delivered dose (*p*<0.05) from the VHC. Lowering the air flow rate also resulted in a lower delivered dose at the VHC outlet, however this was only a trend. (Table 2).

**Table 2.** Delivered Dose ex-VHC in Percentage of Label Claim as a Function of Flow Rate, Volume, and Delay

*Delay (s)*	*Flow rate (L/min)*	*Volume (L)*	*Delivered dose (% of label claim)*	*Standard deviation*
0	10.0	1.00	53.8	9.1
0	10.0	0.25	29.9	1.3
0	5.0	1.00	40.9	8.3
0	5.0	0.25	29.8	2.8
5	10.0	1.00	33.0	4.2
10	10.0	1.00	21.6	3.7

All values listed represent the average out of 10 actuations (represents 5 doses of 5 μg

Tiotropium. 5 μg delivered as 2 puffs of 2.5 μg) of the Respimat^®^. The standard deviation is calculated from two or three repetitions of the experiments, respectively.

Using the age-specific inhalation flow profiles, the delivered doses from the VHC were measured, and are depicted as a percentage of the label claim (5 μg daily dose, 2.5 μg tiotropium per actuation) in Table 3. The measured delivered dose to the filter was 20.3±3.6%, 56.4±2.3%, 64.3±0.6%, and 65.6±4.5% of the label claim (5 μg) for our models of children 0–<2 years, 2–<3 years, 3–<4 years, and 4–<5 years, respectively. In keeping with the experimental setup, there was no delay between the release and the start of the inhalation. An age-dependent increase in the calculated delivered dose was seen for children aged 1–<3 years, which stabilized for children aged >3–≤5 years.

**Table 3.** *In vitro* Determination of Dose of Tiotropium Delivered to Filter/Impactor and to Lung as Function of Age Group

			*FPDTL comparison between age groups*					
*Dataset*			*2–<3*		*3–<4*		*4–<5*	
*Age groups*	*Filter Dose (SD)*	*FPDTL (SD)*	*FPDTL (SD)*	P *Value*	*FPDTL (SD)*	P *Value*	*FPDTL (SD)*	*P Value*
0–<2	20.3±3.6	5.1±1.1%	15.6±1.4%	0.0007	17.9±1.5%	0.0005	37.1±1.8%	0.0001
2–<3	56.4±2.3	15.6±1.4%			17.9±1.5%	0.12	37.1±1.8%	0.0001
3–<4	64.3±0.6	17.9±1.5%					37.1±1.8%	0.0001
4–<5	65.6±4.5	37.1±1.8%						
Adult	N/A	64.9±2.2%						

Delivered dose ex-holding chamber gained with tidal flow profiles as a function of the age group=Filter dose.

FPD *ex throat model* obtained with representative flow profiles for four age groups of children and for adults.

%=percentage of a label claim of the drug, which corresponds to 2.5 μg tiotropium per actuation (two actuations of Respimat^®^ SMI used to deliver 5 μg of tiotropium).

FPDTL, fine-particle dose (aerodynamic diameter <5 μm) to the lung (output of the throat model); SD, standard deviation.

Similarly, the measured FPDTL increases with the age of the child (Table 3). FPDTLs were 5.1±1.1%, 15.6±1.4%, 17.9±1.5%, and 37.1±1.8% of the label claim for children 0–<2 years, 2–<3 years, 3–<4 years, and 4–<5 years, respectively (Table 4). The increase in FPDTL from 15.6% to 17.9% which represents age groups 2–<3 and 3–<4 years, respectively, was not statistically significant (*p*=0.12), because compared to a linear interpolation 15.6% is above and 17.9% is below the interpolated line. The differences in FPDTL between all other age groups were statistically significant (*p*=0.001).

**Table 4.** Conversion From the Experimental Dose-to-the-Lung to the Dose per Body Weight In Vitro Determined by the Throat Models

*Patient* N	*Sex*	*Age (years)*	*Height (cm)*	*Weight (kg)*	*Mouth-Throat V of model (L)*	In vitro *FPD to the lung (% of label dose)*	In vitro *FPD to the lung (μg tiotropium)*	In vitro *(μg/kg)*
22	m	1.4	77	10	0.011	5.1	0.26	0.026
114	f	2.4	91	13.1	0.015	15.6	0.78	0.060
119	m	3.1	101	16.5	0.025	17.9	0.90	0.054
148	m	4.9	114	22.4	0.040	37.1	1.86	0.083

F, female; FPD, fine particle dose; m, male; N, number; V, volume.

The delivered dose to the filter at the output of the VHC and the FPDTL differ considerably, indicating the importance of the dead volume. This volume was minimal in the filter setup but present when using the mouth–throat models. For comparison, the FPD to the lung for an adult, obtained from an idealized model (Alberta mouth–throat model) using the Respimat without a VHC (adult inhalation flow profile and mouth–throat model^(18)^ [and internal study]), was by a factor between two and four higher compared with the doses in the lung experimentally measured for children in the age groups between 2 and <5 years (Table 3).

### Comparison of *in vitro* data with mathematical modeling data on FPD from the VHC to the lung

**FIG. 3.**
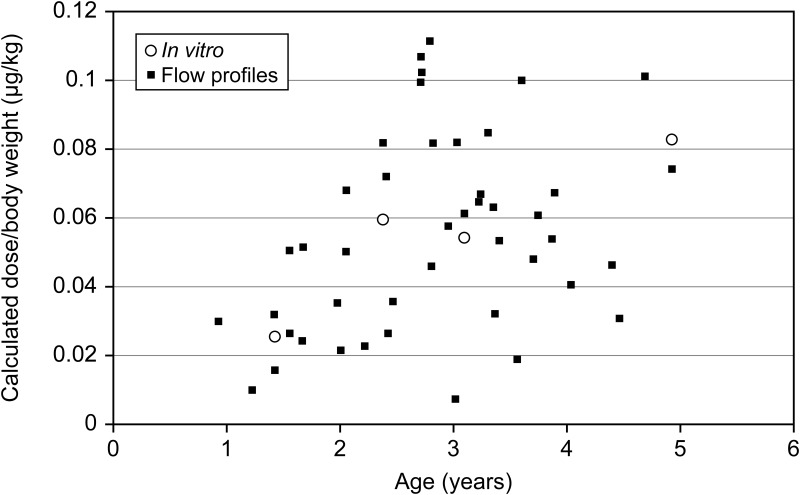
Scatter plot of the calculated dose to the lung per body weight (μg/kg) as function of the age. The graphical representation of the model calculation displays the estimated FPDTL per kg body weight. The *squares* were calculated based on the air flow profiles of each subject, the age (estimating the dead volume), and the body weight of the individual subject. Only values larger than zero were considered. The *open circles* represent the experimental results using the selected inhalation flow profiles and the throat models.

The present assessment is based on the predicted FPDTL. The calculation of the FPDTL shows that the dead volume of the face mask, mouth, throat, larynx, and trachea has a strong impact. The comparison of the *in vitro* data with the model calculations based on inhalation flow profiles shows that the mathematical model operates as expected. The individual children with their flow profiles generate considerable scatter of the predicted dose and form a cloud around the *in vitro* data. The trend of low dose at low age cannot be fully compensated by considering body weight. (Fig. 3).

Grouping these results into the different age group categories generates the subject-based average values of fine particle dose to the lung and fine particle dose to the lung per body weight (Table 5). The calculated *in vitro* dose to the lung (μg/kg [SD]) was 0.031 [0.014], 0.066 [0.031], 0.058 [0.024], and 0.059 [0.029] for children aged 0–<2 years, 2–<3 years, 3–<4 years, and 4–<5 years, respectively.

**Table 5.** Calculated Data for the Dose to the Lung per Kg Body Weight for Various Age Groups

*Age group (years)*	*Estimated average dose to the lung (μg of tiotropium)*	*Average body weight of subgroups (kg)*	*Calculated* in vitro *dose to the lung per body weight (μg/kg)*	*Standard deviation (μg/kg)*
0–<2	0.34	11.0	0.031	0.014
2–<3	0.92	13.9	0.066	0.031
3–<4	0.87	15.0	0.058	0.024
4–<5	1.20	20.3	0.059	0.029
Adult^a^	3.2	70.0	0.046	

^a^ The adult value was determined with the Respimat^®^ SMI connected directly to the throat model.

### Validating the model with obtained PK parameters

In order to validate and test the calculations arising from the mathematical model, these results were compared with PK parameters obtained from a phase 3 tiotropium trial in which urine samples from a subset of children <5 years old with CF were taken.

**FIG. 4.**
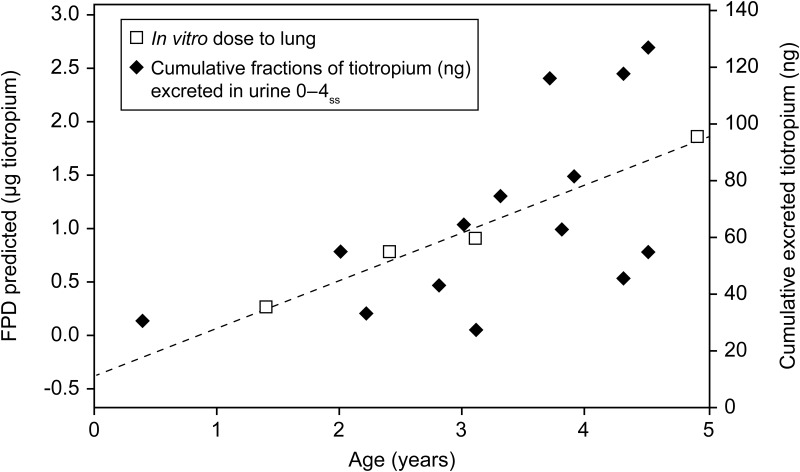
Comparison of the predicted fine particle dose of tiotropium delivered to the lung from the *in vitro* model, and the amount of tiotropium excreted in urine over 4 hours at steady state, R^2^=0.35.

The fraction of the administered tiotropium dose that was eliminated unchanged in the urine over 4 hours following the administration of a single dose (geometric mean: 0.40%; geometric CV: 107%) and at steady-state (geometric mean: 1.19%; geometric CV: 52.9%) demonstrated that systemic exposure was achieved in this age group (Table 6). Based on the amount of drug excreted unchanged over 4 hours post-dose at steady state (Ae_0-4h_[ss]), multiple dosing to steady state resulted in a 3.11-fold (geometric mean ratio) accumulation compared with administration of a single dose of tiotropium 5 μg (Table 6). Furthermore, there was an age-dependent increase in the amount of tiotropium eliminated unchanged in children aged <1–5 years after single and multiple inhaled administrations of 5 μg tiotropium (Fig. 4).

**Table 6.** Summary of pK Parameters of Tiotropium 5 μg After Single and Multiple Inhaled Administrations to Children with CF

*Parameter*	*N*	*Geometric mean*	*Geometric CV (%)*
fe_0–4h_ (%)	13	0.40	107
fe_0–4h(ss)_ (%)	14	1.19	52.9

fe_0–4h,_ fraction of tiotropium eliminated in urine at 0–4 hours; fe_0–4h(ss),_ fe_0–4h_ at steady state; N, number of children.

Figure 4 depicts the comparison of the *in vitro* modeling data to the clinical PK data observed in children aged <5 years with CF. The predicted dose delivered to the lung from the *in vitro* model shows a linear correlation (R^2^=0.35) with the fraction of the observed initial tiotropium dose excreted in urine. The amount excreted in urine parallels the predicted drug exposure, validating the predicted calculated results (Fig. 4). Thus, by calibrating the numerical model and using the anchor points measured according to the mouth–throat models and age group-specific inhalation flow profiles, the information on the scatter of the dose prediction due to age and breathing pattern can be obtained.

## Discussion

Herein, we report on the development and validation of an algorithm that allows for the prediction of the tiotropium dose to the lung in children aged <5 years using the Respimat inhaler equipped with a VHC and face mask.

In order to develop our predictive *in vitro* model for drug delivery to the lung, we combined inhalation flow profile parameters that were representative of children aged 0–<2 years, 2–<3 years, 3–<4 years, and 4–<5 years with corresponding mouth–throat geometries of children in these different age groups. The patients' inspiratory volume and flow rate were derived from the inhalation flow profiles.^(19)^ As recommended by the manufacturer of the VHC, the children performed tidal breathing and were told to inhale for another five breaths after the simulated actuation of the device. The *in vitro* model also takes into account differences in the dead volume between the mouth region and the face mask of the VHC and, most importantly, the difference in the internal throat volume among the four age groups.

The throat volume is one of the key factors in determining how much of the drug is delivered to the lung, and in our studies we find that there is an age-dependent increase in throat volume and the corresponding amount of drug delivered to the lung in the four age groups (Table 2). While the mouth–throat volume increases with age, the stronger growth of the lung volume overcompensates for the effect of the throat and in older children more aerosol is transported to the lungs.

To translate the results to the clinical arena, the *in vitro* data on the predicted dose to the lung were compared with the measured levels of tiotropium in children with CF enrolled in a phase 3 tiotropium clinical trial. Quantitative determination of the dose delivered to the lung in children is challenging because there are limited possibilities to use PK methods in young children, and ethical restrictions on administering ionizing radiation to children for obtaining scintigraphic pictures. Furthermore, due to the difficulty of blood sampling in young children, clinical trials with tiotropium use urine sampling to quantify tiotropium exposure, which is suitable because 74% of the intravenously administered dose is excreted unchanged in urine.^(20,21)^

This makes quantification of tiotropium excreted in urine a good indicator of the dose delivered to the patient's lungs. However, urine sampling presents its own challenges, especially in children aged <1 year. Our studies showed that there is a linear increase with age, in the amount of tiotropium excreted unchanged in urine from the clinical study, the dose to the lung predicted using our model, and the dose to the lung measured in our *in vitro* studies (Fig. 4). This was particularly true for children aged 1–<4 years. Thus, using our model, now validated with the clinical PK data, it should be possible to estimate the dose to the lung based solely on inhalation flow profiles and *in vitro* determination of lung deposition.

A limitation of our study is that the mathematical model assumes that delivery of aerosolized tiotropium in very young children (aged <1 year) is entirely through the mouth. While this is correct for an adult population, children are obligatory nose breathers until age 18 months. According to Chua et al.,^(22)^ delivery of aerosol through the nose to the lower airways is less effective than through the mouth. Moreover, aerosol deposition is influenced by children's breathing pattern and is also correlated with their body weight and age.^(23,24)^ High respiratory rate with variable inspiratory flow, low tidal volume, and smaller airway diameter can diminish deposition of inhaled aerosols to the lower airways in children,^(25)^ resulting in systemic exposure in younger children.^(26)^ This may explain the difference between the predicted dose to the lung and the urinary excretion of tiotropium seen in children aged <1 year in Figure 4, for which data from only one patient were available.

Furthermore, in the younger age groups, improvements of the drug delivery procedure are limited by the dead space in the air duct delivering the medicinal aerosol to the patient. This dead space can be minimized by the choice of the smallest possible face mask available, suitable design of the VHC, and correct handling. Training of the parents as first-line caregivers is essential for the successful treatment of the youngest patients.

The developed mathematical model allows the prediction of doses to the lung and estimation of average doses related to the specific age groups. Based on these theoretical predictions, we showed that children aged <5 years can obtain doses to the lung ranging from 0.031 to 0.066 μg/kg, by using the Respimat SMI equipped with the VHC with face mask. Based on the PK data from clinical studies and the assessment of the flow profiles, it is recommended that children aged <5 years use Respimat with a VHC. We accept that the data provided are not exchangeable for clinical trial data but can support, for example, dose selection to initiate the required clinical development of a drug-device system in children. To assess efficacy and safety of any inhalation product, data from appropriate clinical trials must be obtained. Furthermore, for non-Respimat inhalers, inhalation profiles will need to be generated for the inhaler of choice.

In conclusion, flow profiles obtained from handling studies and the *in vitro* investigation carried out make the mouth–throat model a good predictor of the FPDTL delivery to children via a VHC. The predicted age-dependent trend is confirmed by urinary excretion data. This model can complement clinical studies, and may be a useful tool for the future.

## Supplementary Material

Supplemental data
