# Supplementary material for: In Vitro Determination of Respimat® Dose Delivery in Children: An Evaluation Based on Inhalation Flow Profiles and Mouth–Throat Models
Source: J Aerosol Med Pulm Drug Deliv. 2016 Feb 1;29(1):76–85. doi: 10.1089/jamp.2014.1166 (PMC4739345; doi:10.1089/jamp.2014.1166)
Supplement: Supplemental data [file Supp_Data.pdf]

Supplementary Material

Landmarks determined for selection of typical age-dependent mouth–throat geometries are detailed in Supplementary Figures SF1 and SF2. (Supplementary material is available online at www.liebertpub.com/jamp.)

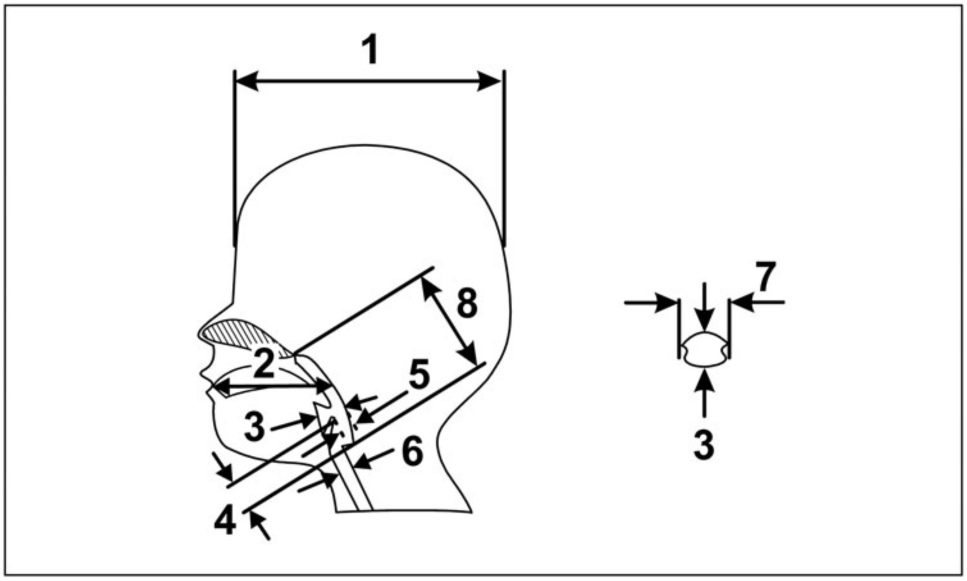

SUPPLEMENTARY FIG. S1. 1. The outer dimension of the head was measured in the sagittal plane from the front to the back. This provided an easily accessible dimension for possible correlations and possibly simple scaling. 2. This dimension is measured from the Lips (*inner side*) to the posterior wall of the Pharynx. When the mouth in the *open position*, this provides the maximum path for an aerosol to propagate until it impacts on the wall of the pharynx. 3. Sagittal distance between the lower back of the tongue and the posterior wall of the Pharynx. 4. Distance from the tip of the Epiglottis (*downwards*) to the onset of the Esophagus. 5. Distance from the tip of the Epiglottis (*backwards*) to the posterior wall of the Pharynx. 6. (*Inner*) diameter of the Trachea. 7. Coronal width of the air duct in the transverse plane located in the middle between Epiglottis and Uvula. 8. Distance from the upper end of the Pharynx (*downwards*) to the onset of the Esophagus.

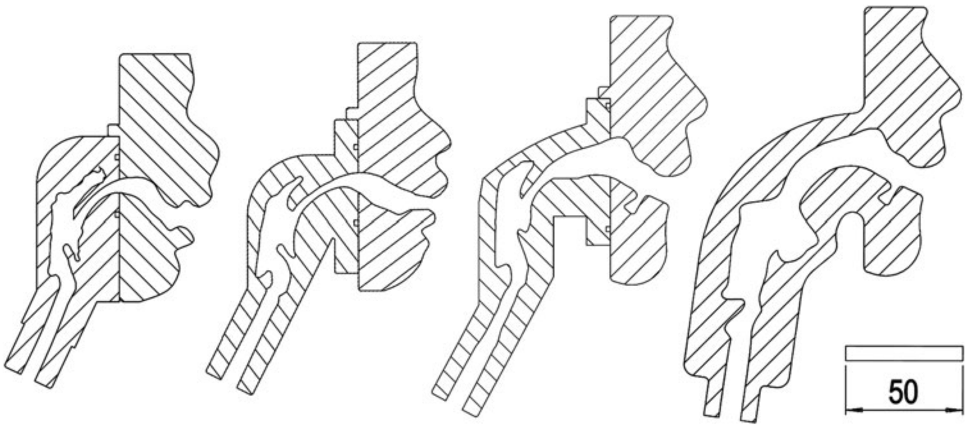

SUPPLEMENTARY FIG. S2. Realistic scaled models of children's extrathoracic airways for *in vitro* inhaler testing. *From left to right*, the models represent children aged 1–2 years, 2–3 years, 3–4 years, and 4–5 years. Bar: 50 millimeters.
